# Peritoneal tuberculosis masquerading as an ovarian malignancy in a young female: A case report

**DOI:** 10.1002/ccr3.8617

**Published:** 2024-03-08

**Authors:** Bright Oppong, Solomon Gyabaah, Gorden Manu Amponsah, Ato Quansah, Eric Amoako Darkwa

**Affiliations:** ^1^ Komfo Anokye Teaching Hospital Kumasi Ghana; ^2^ School of Medicine and Dentistry Kwame Nkrumah University of Science and Technology Kumasi Ghana

**Keywords:** malignancy, masquarading, ovarian, peritoneal, tuberculosis

## Abstract

The clinical manifestations of peritoneal tuberculosis are quite variable, nonspecific and mimic many diseases and pathological conditions such as lymphoma, and ovarian malignancy. Due to this clinical overlap and limited accuracy of diagnostic tests, more awareness of this disease is required to enable early diagnosis and prompt treatment. This is a case of a 25‐year‐old female with no known chronic illness who presented with worsening generalized abdominal pains and distension of 2 months duration. There was an associated significant weight loss of 17 kg. She was initially diagnosed with ovarian malignancy based of ultrasound findings and elevated CA‐125 levels. However, further evaluation later was consistent with peritoneal tuberculosis for which she was treated. Her symptoms resolved completely after 6 months of anti‐tuberculosis treatment. Diagnosis of abdominal TB remains challenging as it is non‐specific. Its features and clinical manifestation overlap with other conditions such as ovarian malignancy. A high index of suspicions and judicious application of the available diagnostic test is need for prompt diagnosis. No single test can effectively diagnose peritoneal TB, but a combination of history, and radiological, immunologic, molecular, and cytologic tests are important.

## 1 INTRODUCTION

In sub‐Saharan Africa, the incidence of tuberculosis is estimated at 212 per 100, 000 population.^1^ In Ghana the prevalence of smear‐positive Tuberculosis (TB) is 111 per 100,000 adult population.^2^ Peritoneal TB is one of the most common manifestations of extra‐pulmonary TB. It forms a common variant of abdominal TB. The incidence of peritoneal TB among all forms of TB varies between 0.1% and 0.7% globally representing 4%–10% of extra‐pulmonary TB and 25%–60% of abdominal TB cases.^3^, ^4^, ^5^ Risk factors include any form of underlying immunosuppression such as human immunodeficiency virus infection, diabetes mellitus, treatment with anti‐tumor necrosis factor (TNF) agents, ongoing peritoneal dialysis and hepatic cirrhosis, underlying malignancy, chronic steroid use and malnutrition.^3^, ^6^ The clinical manifestations of peritoneal TB are quite variable, and nonspecific and mimic many diseases and pathological conditions such as lymphoma, and ovarian malignancy and thus require a high index of clinical suspicion to avoid delayed diagnosis with its associated morbidity and mortality.^7^ Clinical manifestations include abdominal pain and swelling, fever, night sweats, weight loss, ascites, and hepatomegaly. Overlap of symptoms with other disease conditions and limited accuracy of diagnostic tests demand more awareness of this disease to enable early diagnosis and prompt treatment. We describe the case of a 25‐year‐old female who presented with symptoms and findings that were initially consistent with an ovarian malignancy but were later found to be a peritoneal TB.

## 2 CASE HISTORY AND EXAMINATION

A 25‐year‐old woman presented to the outpatient department of the Komfo Anokye Teaching Hospital in January 2023 with the chief complaint of worsening generalized abdominal pains and distension 2 months duration.

She had lost around 17 kg weight in the preceding 5 months. There were no symptoms of fever, chronic cough, hemoptysis or night sweats. She had been well before these complaints started and had no known prior history of any chronic illness or previously diagnosed tuberculosis. There was also no known family history of malignancies and no known close contact with tuberculosis or chronic cough. She previously visited another hospital a month before presenting to our hospital with the same complaints where an ultrasound scan showed a suspicion of ovarian malignancy with septations and irregularities and she was subsequently referred to our hospital for further management.

Physical examination on her first presentation to our hospital showed normal vital signs with a blood pressure of 122/79 mmHg, respiratory rate of 16 cycles per minute, heart rate of 98 beats/min, and temperature of 36.6°C. Additionally, oxygen saturation was 93% breathing ambient air. The chest was clear clinically, and cardiac auscultation was normal. Abdominal examination showed the presence of moderate ascites. No peripheral edema was observed. The rest of the physical examinations were normal.

## 3 METHODS

Complete blood count and other laboratory examinations on this first visit revealed low MCV anemia with 8.1 g/dL of hemoglobin, slightly elevated lymphocyte percentage, erythrocyte sedimentation rate (ESR) was 95 mmfall/h, normal aspartate transaminase (AST) and alanine transaminase (ALT) as well as a reduced albumin level. The cancer biomarker result which was available 7 days later showed elevated CA‐125 of 792.1 U/mL.

**FIGURE 1:**
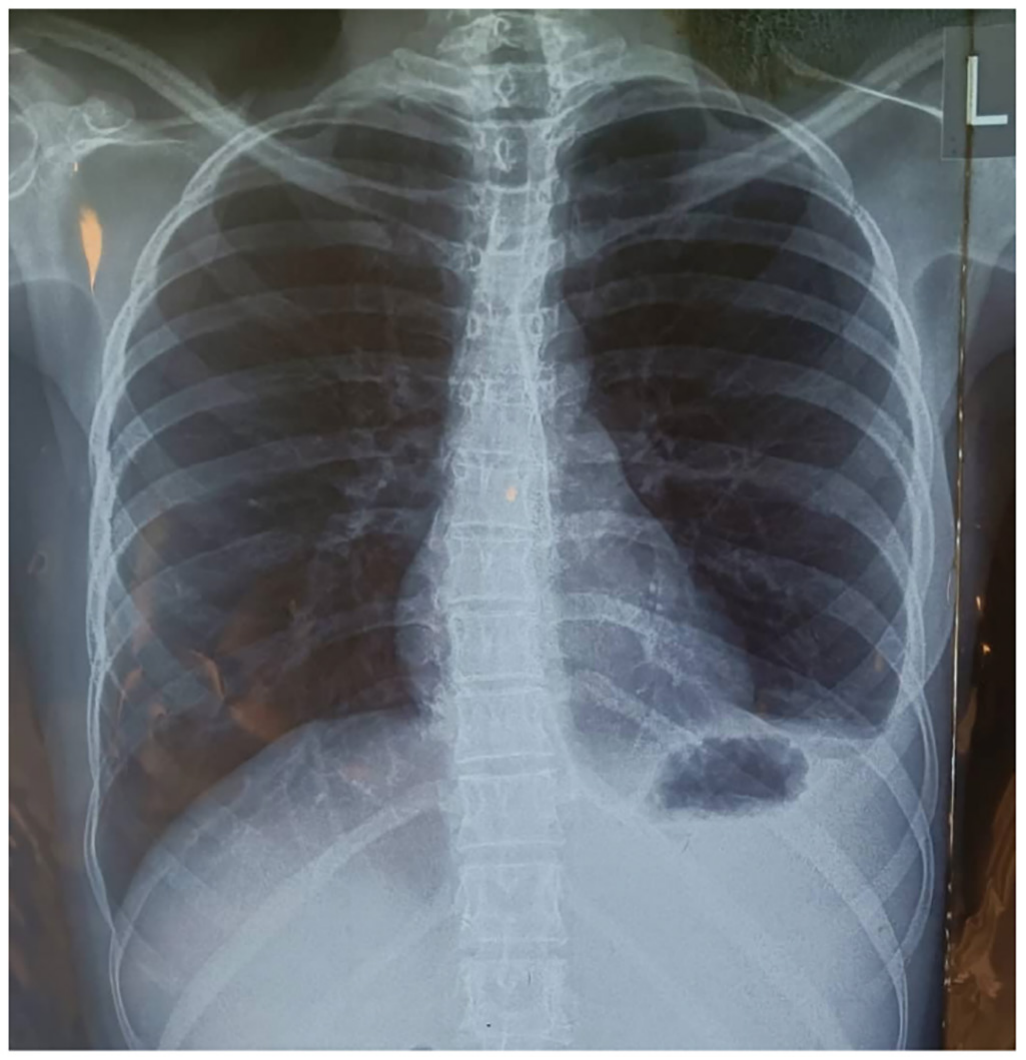
Initial chest x‐ray showed clear lung fields with minimal left pleural effusion.

Abdominal paracentesis done 2 days later aspirated 540 mL of serous ascitic fluid. Microbiology analysis of ascitic fluid showed cells that were predominantly lymphocytes with a cell count of 300 cells/mm^3^. No organism was cultured. Cytology of the fluid did not reveal any malignant cells. No bacteria was detected on the gram stain. Gene Xpert test on the ascitic fluid did not detect *Mycobacterium tuberculosis (MTB)*. The serum ascitic acid albumin gradient was 0.98 g/dL suggestive of exudative ascitic fluid. The ascitic fluid was then examined for adenosine deaminase (ADA) and the result was 54.6 IU/L (tuberculosis if ADA > 40), which indicated that the cause of the ascites was likely to be TB. Her initial chest radiograph (CXR) on the day of presentation showed clear lung fields with minimal left pleural effusion (Figure 1).

**FIGURE 2:**
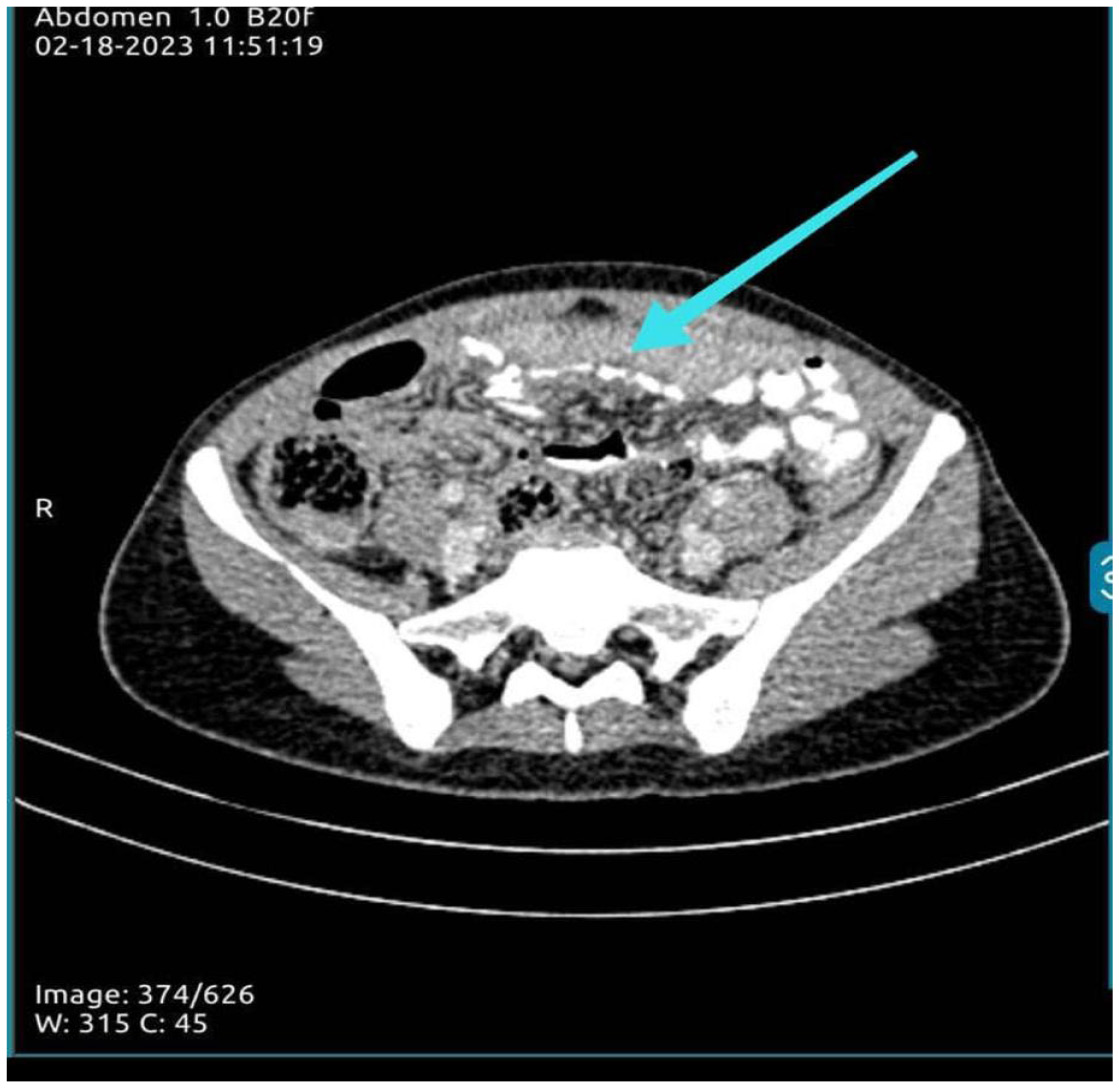
Cross‐sectional abdominal CT scan image showing evidence of diffuse peritoneal thickening with diffuse thickening of the small bowels with multiple nonspecific enlarged lymph nodes.

Contrast‐enhanced CT scan of the abdomen taken 2 weeks later showed evidence of diffuse peritoneal thickening with diffuse thickening of the small bowels, and multiple non‐specific enlarged lymph nodes in the retroperitoneum measuring 1.2 cm on average. Minimal fluid was noted in the pelvis and the perihepatic/perigastric region with enhancing wall/periphery. The liver was diffusely enlarged with a span of 19.3 cm with a smooth contor and no focal lesions. The Gallbladder was adequately distended. The liver, gallbladder, spleen, pancreas, adrenal glands, kidneys, uterus and ovaries were unremarkable. The colon was moderately thickened and distended but not obstructed. These abdominal CT scan findings were highly suggestive of tuberculous peritonitis. The patient's quantiferon TB gold test (IGRA) was positive with a value of 19.4 IU/mL. The patient refused peritoneal biopsy. Figure 2

## 4 CONCLUSION AND RESULTS

Considering the patient's presentation and the limited available laboratory and radiological results, a diagnosis of peritoneal TB was of high suspicion and thus a decision was taken to start the patient on an anti‐tuberculosis drug (Oral isoniazid 300 mg daily Oral rifampicin 600 mg daily, Oral pyrazinamide 1.5 daily, Oral ethambutol 1200 mg daily) for 2 month and then rifampicin and isoniazid was continued at the maintenance phase for 4 months. She was monitored for resolution of symptoms and drug toxicity.

At 5 months on anti‐TB treatment, the patient had resolution of all her symptoms and had no significant side effects of the anti‐TB medications. Her ascites had almost resolved. CA‐125 level decreased to 10.75 units/mL. The patient completed 6 months of the anti‐TB medications.

A repeat abdominal CT scan showed that the thickening of the colon had resolved. The liver span had reduced to 12 cm. The peritoneal thickening had resolved, retroperitoneal lymph nodes were now of normal sizes and other abdominopelvic organs were all of normal morphology except for an incidental follicular cyst. Currently patient is doing much better with no complaints.

## 5 DISCUSSION

The underlying mechanism for tuberculous peritonitis is varied. First, it can occur due to the reactivation of latent foci established in the peritoneum through a hematogenous spread to the mesenteric lymph node from a primary pulmonary infection.^8^ Thus, TB peritonitis is usually a secondary infection from the spread of the tubercle from a pulmonary focus into the peritoneum. Another important mechanism is the ingestion of the bacilli through sputum and the subsequent spread of the organism through the Payer's patches in the intestinal mucosa to the mesenteric lymph nodes.^8^ There can also be contiguous spread from the intestine and the fallopian tubes. Tuberculous peritonitis is traditionally divided into three types depending on the nature, amount and characteristics of the ascitic fluid produced.^9^ The first type is the “wet” type, the most common and is associated with large amounts of ascitic fluid that may be either diffusely distributed or loculated. The second is the “fibrotic‐fixed,” less common and characterized by omental masses, matted loops of bowel and mesentery and occasionally loculated ascites. Lastly, the “dry‐plastic” type is uncommon and consists of caseous nodules, fibrous peritoneal reaction and dense adhesions. However, the first two types can overlap each other. Our patient had the first type.

Peritoneal TB has an insidious onset and its symptoms develop over several weeks to months. The symptoms are non‐specific and greatly overlap with other conditions such as ovarian malignancy and abdominal lymphoma. Also, CA‐125 which is used as a screening test for ovarian malignancy may be elevated in peritoneal TB. Due to these overlaps, clinical diagnosis of peritoneal TB is usually a challenge.

In a systematic review of 35 studies, it was shown that abdominal pain is the commonest presenting symptom accounting for 64.5% of the clinical manifestation of abdominal TB followed by weight loss (61%) and low‐grade fever usually with night sweats.^3^ Other signs and symptoms include anorexia, general malaise, abdominal swelling, diarrhea or constipation, ascites and hepatosplenomegaly. Peritoneal TB has no sexual predilection and it is commonly seen in patients between 35 and 45 years of age. Similarly, ovarian malignancy usually presents with abdominal pain, abdominal distension, early satiety, weight loss, low‐grade fever, general malaise and increased urinary frequency and urgency. However, ovarian malignancy is typically found in patients above 50 years even though it can occur in the young less than 40 years of age.^10^ In this report, the patient was a 25‐year‐old female who had an insidious onset of abdominal pain and distension that gradually worsened over 2 months preceded by significant weight loss but no fever or night sweats. Clinical examination did not reveal much aside from ascites which was moderate. These symptoms could easily pass for ovarian or any other abdominal malignancy. Thus, the diagnosis of peritoneal TB was difficult to make based on clinical findings.

Elevated CA‐125 is usually considered highly suspicious for epithelial ovarian malignant tumors, especially in postmenopausal women, and usually reaches very high levels in patients with widespread peritoneal dissemination.^11^, ^12^ However, it is a nonspecific marker and it may also be elevated in other conditions such as endometriosis, endometrial cancer, functional ovarian cyst, pelvic inflammatory disease, uterine leiomyoma, cirrhosis and other liver conditions, colitis, heart failure, nephrotic syndrome, pancreatic malignancy and peritoneal TB. Thus, elevated CA‐125 does not offer any diagnostic advantage in differentiating peritoneal TB from ovarian malignancy. However, in both cases, CA‐125 can be used as a follow‐up marker in ascertaining response to treatment. In this case study, there was a significant decrease in the serum CA‐125 levels after the initiation of anti‐TB treatment. ADA level is an important diagnostic tool for the detection of peritoneal TB. In a systematic review and meta‐analysis involving 3044 participants, the pooled sensitivity and specificity of ADA for abdominal TB detection were 93% [95% confidence interval (CI): 0.89–0.95] and 95% (95% CI: 0.93–0.96), respectively.^13^ Elevated ascitic ADA levels are highly suggestive of peritoneal TB. The patient in this report had an elevated ascitic ADA level which shifted the diagnosis more towards peritoneal TB supported by a positive Quantiferon TB Gold test. Peritoneal biopsy could not be done for this patient because of the patient's refusal which was a major limitation.

A peritoneal biopsy specimen is positive on acid‐fast stain in 50% to 63% of cases, and culture is typically positive in about 70% of cases.^3^, ^14^, ^15^ Histopathological evaluation of the tissue may reveal the presence of caseating granulomas in 70%–95% of patients as an essential diagnostic clue, while PCR of the tissue is positive in 25%–70% of cases.^3^, ^14^, ^15^

An abnormal chest x‐ray may be frequent in a patient with peritoneal TB. In a systemic review by Sanai and Bzeizi, it was shown that abnormal chest X‐ray findings are seen in 19%–83% of cases averaging to about 38% based on cumulative data of over 1000 patients.^3^ Chest x‐ray can however, be normal in situations where the infection is limited to the peritoneum as in the case of our patient. Contrast‐enhanced abdominal CT scan showed evidence of diffuse peritoneal thickening with diffuse thickening of the small bowels, there are multiple non‐specific enlarged lymph nodes in the retroperitoneum measuring an average of 1.2 cm which was highly suggestive of peritoneal TB even such findings may be observed in peritonitis carcinomatosis. The most common CT findings in peritoneal tuberculosis include ascites (70%–90% of cases), smooth peritoneal thickening with marked enhancement, densification of the mesenteric root fat planes and lymph node enlargement with areas of central necrosis or calcification.^16^ Other diagnostic tools or techniques that can be employed in the diagnosis of peritoneal TB include gene Xpert on ascitic fluid and the ligase chain reaction (LCR) DNA amplification methods which are a highly specific test for the detection of *Mycobacterium tuberculosis* but low sensitivity of about 28%.^17^

The recommended treatment for a new patient with peritoneal TB according to the World Health Organization is 6 monthly regimen of anti‐tuberculosis medication involving 2 months of rifampicin, pyrazinamide, isoniazid, ethambutol and 4 months of rifampicin and isoniazid.^18^ There was a tremendous improvement in the patient's condition when she was started on anti‐TB medication. Repeat abdominal CT had almost normal findings except for the follicular cyst which was incidentally detected.

Diagnosis of abdominal TB remains challenging and requires more awareness. Due to its nonspecific features and overlap of clinical manifestation with conditions such as ovarian malignancy, it requires a high index of suspicions and judicious application of the available diagnostic test. No single test can effectively diagnose peritoneal TB, but a combination of history, and radiological, immunologic, molecular and cytologic tests are important. Clinicians need to consider peritoneal TB as a differential diagnosis in patients presenting with abdominal pain and swelling particularly in high TB incidence countries such as Ghana.

### 5.1 Limitation

The study was limited by the absence of tissue biopsy.

## AUTHOR CONTRIBUTIONS

**Bright Oppong:** Conceptualization; data curation; investigation; methodology; project administration; resources; supervision; writing – original draft; writing – review and editing. **Solomon Gyabaah:** Conceptualization; data curation; formal analysis; investigation; methodology; project administration; validation; writing – original draft; writing – review and editing. **Gorden Manu Amponsah:** Data curation; investigation; methodology; validation; writing – original draft; writing – review and editing. **Ato Quansah:** Data curation; investigation; methodology; supervision; validation; writing – original draft; writing – review and editing. **Eric Amoako Darkwa:** Formal analysis; investigation; methodology; resources; supervision; validation; writing – original draft; writing – review and editing.

## FUNDING INFORMATION

There was no funding for this study.

## CONFLICT OF INTEREST STATEMENT

The authors declare no conflict of interest.

## CONSENT

Written informed consent was obtained from the patient.

## Data Availability

Data is available upon reasonable request to the corresponding author.
